# Preparedness of Dental Undergraduates for Clinical Practice: A Comparison Between Evaluations by Students and Academic Faculty

**DOI:** 10.1155/ijod/9065801

**Published:** 2025-02-28

**Authors:** Kamis Gaballah, Kamran Ali, Daniel Zahra, Eteman Ibrahim

**Affiliations:** ^1^Department of Oral and Maxillofacial Surgery, University of Sharjah, Sharjah, UAE; ^2^QU Health College of Dental Medicine, Qatar University, Doha 2713, Qatar; ^3^School of Psychology, Plymouth University, Plymouth PL4 8AA, UK

**Keywords:** dental, preparedness, students, supervisors, undergraduate

## Abstract

**Introduction:** Undergraduate dental education aims to equip students with scientific knowledge, clinical skills, and behavioral attributes to prepare them for a career in independent dental practice after graduation. The purpose of this study was to compare self-reported preparedness of undergraduate dental students with evaluations by their clinical supervisors.

**Methods:** A probability sampling technique was used to recruit final year dental students at a university in the Middle East. Preparedness of the students was assessed using a previously validated dental preparedness assessment scale encompassing 50 core clinical skills (Part A) as well as cognitive attributes and behavioral skills (Part B). The participating students were also evaluated by their clinical supervisors.

**Results:** A total of 52 of the 70 students invited provided their responses, yielding a response rate of 74.28%. In addition, four clinical supervisors also evaluated the preparedness of the participants. The mean student ratings of their preparedness were significantly higher than ratings provided by their supervisors for Part A, *t* (93.13) = 7.48, *p* < 0.001; Part B, *t* (101.46) = 7.25, *p* < 0.001; and overall, *t* (98.48) = 8.25, *p* < 0.001.

**Discussion:** This study compared students' self-perceived preparedness for dental practice with evaluations by their supervisors. The students rated their preparedness to be higher compared to the evaluations by their supervisors, indicating weak correlations between self-perceived preparedness and supervisors' evaluation. Nevertheless, evaluation by supervisors provides a powerful tool for students to compare their self-assessment and reflect on their strengths and weaknesses for work readiness after graduation.

## 1. Introduction

Undergraduate dental education strives to prepare future dentists by fostering scientific knowledge, evidence-based clinical care, competence in clinical procedural skills, professionalism, effective communication, teamwork, reflective practice, and a commitment to lifelong learning [1, 2]. The primary objective of undergraduate dental education is to equip dental students for professional practice, ensuring a seamless transition from university training to independent clinical practice [3, 4]. While dental schools deliver education, training, and assessments for undergraduate students based on core learning outcomes, research indicates that graduates may still exhibit gaps in knowledge and skills upon completing their studies [5–9]. It is crucial for dental schools to evaluate the preparedness of their students consistently throughout the undergraduate program to identify gaps in knowledge and skills, allowing for timely support and remediation [10].

While formative and summative assessments of student performance in individual courses or modules are essential for making informed decisions about student progression, dental educators may also consider conducting holistic evaluations of students' preparedness, aligned with the program's learning outcomes. While formative and summative assessments of student performance in individual courses or modules are essential for making informed decisions about student progression, dental educators may also consider conducting holistic evaluations of students' preparedness, aligned with the program's learning outcomes [11]. Regular assessment of students' readiness for practice can serve as an effective tool to evaluate the success of dental education programs, teaching strategies, curriculum implementation, and students' professional development. By benchmarking individual student performance, educators can pinpoint areas requiring further reinforcement and take informed steps to ensure graduates are prepared to deliver safe and effective dental care independently. Addressing these gaps during the university program is crucial, as students may face greater challenges in seeking assistance for deficiencies once they leave the academic environment [4, 12].

This study aimed to compare self-reported preparedness of undergraduate dental students with evaluations by their clinical supervisors.

## 2. Methods

### 2.1. Research Ethics

The University of Sharjah, Research Ethics Committee, approved the survey (approval number REC-23-03-28-01-F). Participation in the survey was voluntary and the information collected was not used to identify respondents.

### 2.2. Study Design

An analytical cross-sectional study design was employed for this study. The data was collected from 1st March to 30th May, 2023.

### 2.3. Settings

The research was conducted at the College of Dental Medicine at the Sharjah University.

### 2.4. Research Instrument

This study utilized the Dental Undergraduate Preparedness Assessment Scale (DU-PAS) which is a reliable and validated instrument for evaluating preparedness of dental students and new graduates and has been used in multiple studies [13]. DU-PAS consists of a 50-item inventory divided into two parts. Part A has 24 items to evaluate clinical skills, while Part B has 26 items to evaluate knowledge and affective skills. All items in Part A are scored on a 3-point scale which are scored as follows: no experience (0), with verbal and/or practical input from a colleague (1), and on my own, independently (2). All items in Part B are scored on a 3-point scale as follows: no experience (0), mostly (1), and always (2). Therefore, the total maximum score for the 50-item inventory is 100.

### 2.5. Participants and Sampling Technique

A non-randomized convenience sampling technique was used to target undergraduate students in Year 5 (final year) of the Bachelor of Dental Surgery (BDS) programme. The eligibility criteria was based on full-time dental students in Year 5 of the BDS programme. Students who had interrupted their studies were excluded.

Invites to participate in the research were sent using the institutional e-mail of 70 undergraduate students, of which 52 responded (yielding a response rate of 74.28%). In addition, the clinical supervisors of the students were also invited to score the students using the same research instrument, that is, DU-PAS.

### 2.6. Data Collection

Participants provided their responses to DU-PAS independently. The students provided their self-perceived preparedness using electronic tablets in a face-to-face session during the second semester of the academic year. The supervisors completed student evaluations for individual students separately.

### 2.7. Data Analysis

Data analysis was conducted using the R [14] statistical environment for Windows. Sample and subgroups distributions of scores for each part of the DU-PAS were computed. The distribution of categorical response options between groups were compared using *χ*^2^ tests of association. Analysis of variance (ANOVA) and independent-samples *t*-tests were used for analyzing numeric responses. Pearson's correlation and Cohen's kappa coefficients were used to evaluate interrater reliability and relationships between scores for each item within subgroups.

## 3. Results

The proportion of this sample by gender and nationality are shown in Table 1. All participants were Year 5 BDS students aged 20–25 years. The participating students were also scored by four supervisors (A–D). The number and gender of students evaluated by each supervisor are as follows:

• Supervisor A: 14 (eight females, six males)
• Supervisor B: 8 (seven females, one male)
• Supervisor C: 18 (15 female, three males)
• Supervisor D: 12 (seven females, five males)

### 3.1. Overall Scores

Overall scores by part (A and B) and by source (students and supervisors) are shown in Table 2. These are calculated based on converting the categorical responses to numerical values (no experience = 0, with input/mostly = 1, and independently/always = 2).

Independent-samples *t*-tests showed that the ratings differed significantly between students and supervisors for Part A, *t* (93.13) = 7.48, *p* < 0.001; Part B, *t* (101.46) = 7.25, *p* < 0.001; and total score, *t* (98.48) = 8.25, *p* < 0.001.

**Figure 1:**
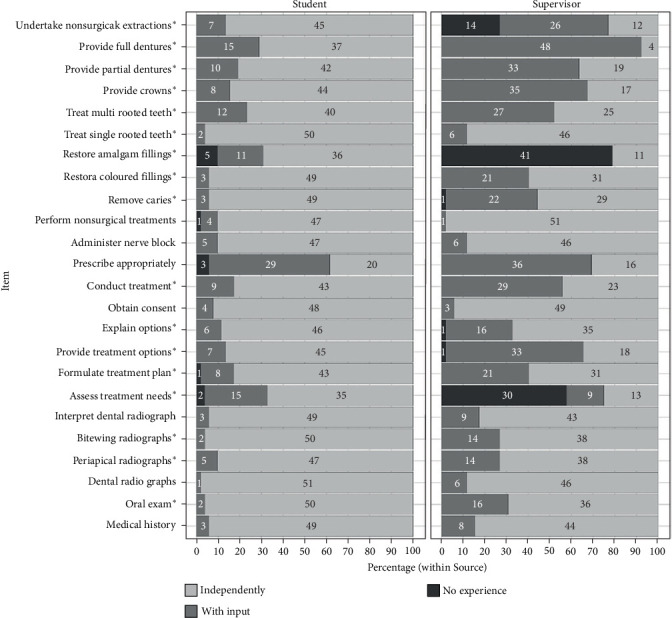
Distributions of scores for each item in Part A given by students and supervisors. *⁣*^*∗*^Chi-squared tests for items which showed a statistically significant variation in the distributions between students and supervisors at the *p* < 0.05 level are starred.

**Figure 2:**
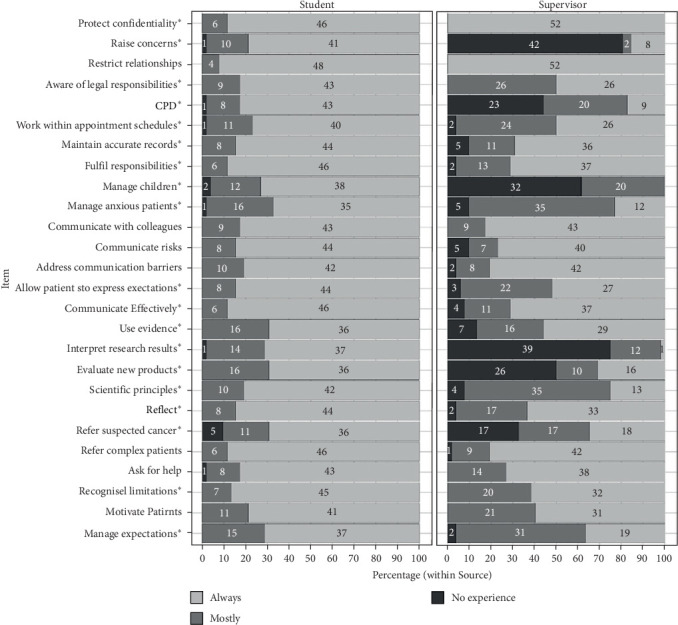
Distributions of scores for each item in Part B given by students and supervisors. *⁣*^*∗*^Chi-squared tests for items which showed a statistically significant variation in the distributions between students and supervisors at the *p* < 0.05 level are starred.

Figures 1 and 2 showed the distributions of scores for each item in Part A and Part B, respectively. Starred items were items for which *χ*^2^ tests showed a statistically significant variation in the distributions between students and supervisors at the *p* < 0.05 level.

### 3.2. Correlations Between Items

**Figure 3:**
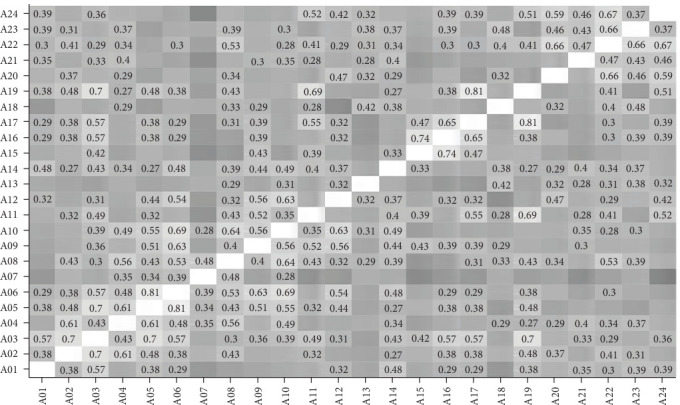
Interitem correlations of student ratings for Part A items.

**Figure 4:**
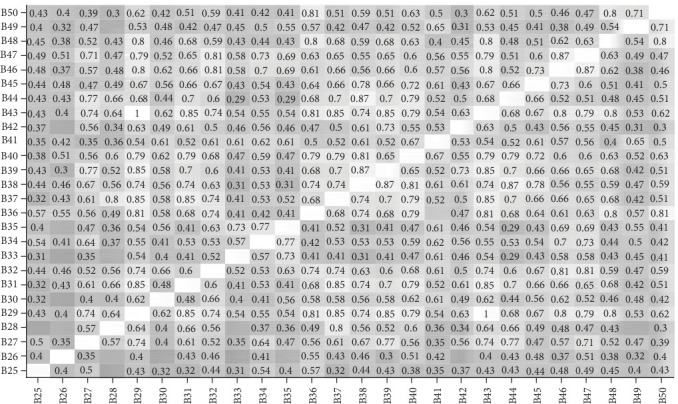
Interitem correlations of student ratings for Part B items.

**Figure 5:**
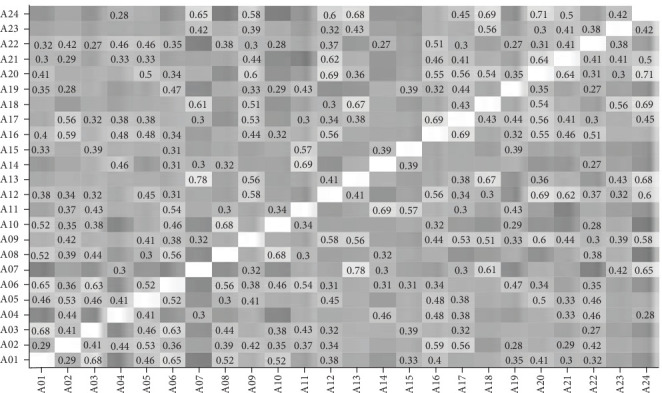
Interitem correlations of supervisor ratings for Part A items.

**Figure 6:**
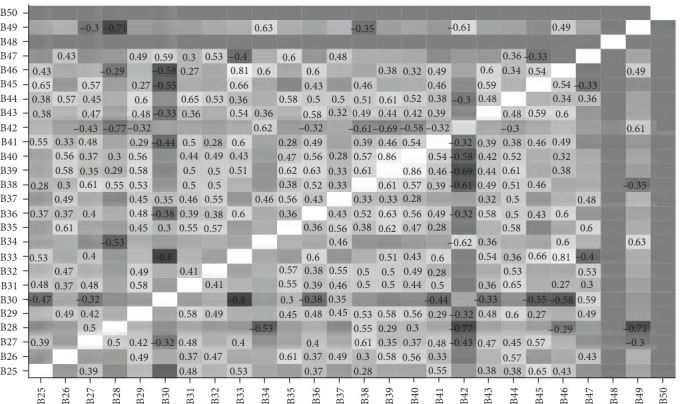
Interitem correlations of supervisor ratings for Part B items.

Figures 3–6 present the correlations between students and supervisors between ratings for items in Part A and Part B. The figures displayed correlation coefficients only for statistically significant relationships (*p* < 0.05), with lighter shades indicating stronger and more positive correlations.

### 3.3. Interrater Agreement Between Students and Supervisors

Across all student–supervisor pairs for Part A items, the mean of Cohen's kappa interrater reliability coefficients was 0.08 (Min = −0.17, Max = 0.55, SD = 0.14), suggesting agreement ranges from “none” to “slight” (using equal difference weightings, and based on accepted thresholds for interpretation) [15].

Converting Part A item responses to numerical values (no experience = 0, with input = 1, and independently = 2), the correlation between student and supervisor ratings was *r* = 0.16; *t* (1246) = 5.68, *p* < 0.001; a small correlation by Cohen's thresholds [16].

Across all student–supervisor pairs for Part B items, the mean of Cohen's kappa interrater reliability coefficients was 0.06 (Min = −0.14, Max = 0.40, SD = 0.13), suggesting agreement ranges from “none” to “slight”, with similarly low correlation observed between student and supervisor ratings; *r* = 0.07, *t* (1350) = 2.58, *p*=0.01 when responses were converted to a 0–1–2 numerical scale.

**Figure 7:**
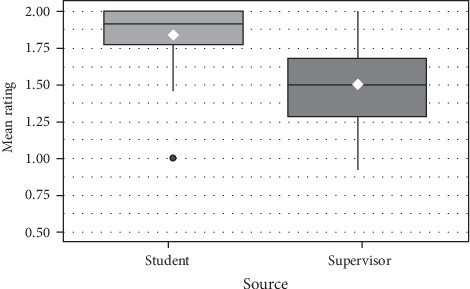
Distribution (boxplots) and means (white diamond) of mean ratings for Part A items by students and supervisors.

**Figure 8:**
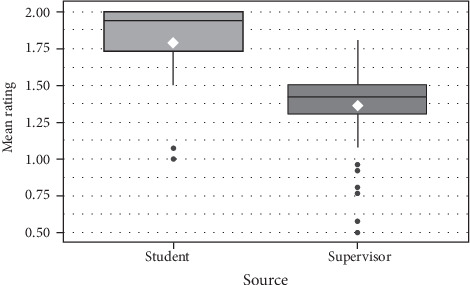
Distribution (boxplots) and means (white diamond) of mean ratings for Part B items by students and supervisors.

Distribution of mean ratings for each item by students and supervisors for Part A and Part B items are shown in Figures 7 and 8 respectively.

### 3.4. Differences Between Supervisors

A *χ*^2^ test of association suggested some variation between supervisors and their distributions of ratings for Part A items (*χ*^2^ = 105.49, *df* = 6, *p* < 0.001). This seemed to be driven by Supervisor B awarding a proportionally higher number of “independent” ratings. This difference by supervisor was replicated when treating the responses as numeric (no experience = 0, with input = 1, and independently = 2); *F*_(3,1244)_ = 28.32, *p* < 0.001.

The same analysis also suggested some variation between supervisors and their distributions of ratings for Part B items (*χ*^2^ = 150.31, *df* = 6, *p* < 0.001). This seemed to be driven by Supervisor B awarding a proportionally higher number of “always” ratings and is again replicated when treating the responses as numeric (no experience = 0, mostly = 1, and always = 2); *F*_(3,1348)_ = 41.27, *p* < 0.001. Ratings by supervisors for Part A and B are shown in Table 3.

## 4. Discussion

DU-PAS has been used in multiple studies to evaluate readiness of dental students and new graduates for clinical practice [17–20]. However, the previous studies have only reported on self-perceived preparedness of students and new graduates. The main limitation of the previous studies was that self-assessment may not truly reflect the actual preparedness of the students and new graduates.

This is the first study, to the authors' knowledge, to collect data simultaneously from students and their supervisors to allow a direct comparison between self-perceived preparedness with evaluations by the supervisors. Statistically significant differences were observed between the mean scores by students and supervisors for both Part A and Part B of the DU-PAS as well as the total scores. Given that the student scores were not corroborated by supervisor evaluations shows that the student participants in the current study may have overestimated their preparedness. The results of the current study corroborate with previous work with students in healthcare professions, which have shown poor correlations between students' self-reported competence and assessment by their supervisors [21–23]. In line with the recommendations in the medical education literature, the current work analyzes self-assessment as both a correlation and a paired comparison with supervisor scores [21].

The self-perceived ratings of student participants in this study were higher than those observed for students from the United Kingdom, Malaysia, Pakistan, and Saudi Arabia [17–20]. However, the scores by supervisors in the current study are comparable to those reported in the aforementioned countries. Although we collected demographic data of the participants, small numbers of students of each nationality, and uniformity of age and stage of study precluded any meaningful analysis of these factors.

Encouraging students to undertake a self-assessment of their readiness for dental practice enables them to evaluate their performance comprehensively against the program learning outcomes. It also encourages them to look ahead to appreciate what is expected from them as dental graduates, while enacting their professional identity [22, 23]. In addition, simultaneous evaluation by supervisors provides a powerful tool for students to compare their self-assessment and reflect on their strengths and weaknesses.

Notwithstanding the disparities in student and supervisor scores in the current study, a key area of weakness identified by both the students as well as supervisors related to the ability to prescribe medications appropriately. Dentists frequently prescribe a range of medications and it is a core skill. Inadequate prescribing skills amongst medical and dental students has been highlighted in the literature [24–26]. The findings may prompt dental educators to reflect on current approaches to teaching prescribing and consider ways of improving student proficiency and preparedness in this domain.

Supervisors evaluations also identified areas where a large number of students lacked experience including assessment of treatment needs of patients and raising concerns about inappropriate behavior/practice by colleagues. Assessment of treatment needs of patients requires a person-centred approach and a consideration of patients' social, cultural, and ethnic background as well as expectations from treatment [27]. With rapid globalization, urban populations are becoming increasingly multicultural and education on cultural competence needs to be considered in undergraduate dental curricula [28]. Institutions also need to have clear policies on raising concerns which should be shared with the students and staff and supported with periodic training. Other weaknesses identified by the supervisors related to lack of students' experience in interpreting results of research studies and evaluating new dental materials and products. A comprehensive approach is recommended for teaching research skills and evidence-based dentistry, combining formal training in critical literature appraisal with the effective integration of modern technologies, including mobile devices, simulations, and online resources [29].

The main limitation of this study is that the data was collected from a single institution and future multi-institutional studies may be required to further explore the correlations between self-assessment of preparedness by the students and their supervisors. Moreover, obtaining evaluations of each student from two or more supervisors may be beneficial in avoiding any impact of assessor variation, but this was not possible in the current study due to resource constraints. Nevertheless, this may be considered in future studies. Given the paucity of published studies exploring these correlations in dental education, the current study adds value to the literature and may provide impetus to dental academics to explore this further.

## 5. Conclusion

This study compared students' self-perceived preparedness for dental practice with the evaluations by their supervisors. The students rated their preparedness to be higher compared to the evaluations by their supervisors, indicating weak correlations between self-perceived preparedness and supervisors' evaluation. Nevertheless, evaluation by supervisors provides a powerful tool for students to compare their self-assessment and reflect on their strengths and weaknesses for work readiness after graduation.

## Figures and Tables

**Table 1:** Proportions of students in sample by nationality and gender.

Nationality	Frequency			Percentage		
	Female	Male	Total	Female	Male	Total
A	0	1	1	0.00	6.67	1.92
B	1	0	1	2.70	0.00	1.92
C	1	1	2	2.70	6.67	3.85
D	2	4	6	5.41	26.65	11.54
E	5	1	6	13.51	6.67	11.54
F	5	6	11	13.51	40.00	21.15
G	8	0	8	21.63	0.00	15.38
H	1	1	2	2.70	6.67	3.85
I	2	0	2	5.41	0.00	3.85
J	5	1	6	13.51	6.67	11.54
K	5	0	5	13.51	0.00	9.61
L	2	0	2	5.41	0.00	3.85
Total	37	15	52	—	—	—

**Table 2:** Descriptive statistics for overall scores by Part (A and B) and source (student and supervisor).

Part	Source	*N*	Mean	SD	Min.	Max.	Range	IQR
A (24 items)	Students	52	44.19	4.55	24	48	24	5.25
	Supervisors	52	36.17	6.26	22	48	26	9.25

B (26 items)	Students	52	46.63	8.08	26	52	26	7.00
	Supervisors	52	35.54	7.51	13	48	35	5.25

Total (50 items)	Students	52	90.83	10.63	63	100	37	13.25
	Supervisors	52	71.71	12.88	40	96	56	13.50

**Table 3:** Ratings by supervisors.

Supervisor	Part A				Supervisor	Part B			
	A	B	C	D		A	B	C	D
Frequency									
Independently	168	161	264	128	Always	214	141	280	84
With input	126	31	137	145	Mostly	80	67	117	146
No experience	42	0	31	15	No experience	70	0	71	82
Percentage (within supervisor)									
Independently	50.00	83.85	61.11	44.44	Always	58.79	67.79	59.83	26.92
With input	37.50	16.15	31.71	50.35	Mostly	21.98	32.21	25.00	46.79
No experience	12.50	0.00	7.18	5.21	No experience	19.23	0.00	15.17	26.28

## Data Availability

The data underlying this article will be shared on reasonable request to the corresponding author.
